# Bis(2,6-dichloro­benz­yl)selane

**DOI:** 10.1107/S1600536812008318

**Published:** 2012-02-29

**Authors:** Mei-Yun Zhou, Yi-Qun Li, Wen-Jie Zheng

**Affiliations:** a Department of Chemistry, Jinan University, Guangzhou 510632, People’s Republic of China

## Abstract

The title mol­ecule, C_14_H_10_Cl_4_Se, features a selenide bridge between two dichloro­benzyl units. The dihedral angle between the two benzene rings is 107.9 (16)°. In the crystal, weak π–π face-to-face aromatic inter­actions are observed [centroid–centroid distance between two adjacent (but crystallographically different) phenyl rings = 3.885 (5) Å], providing some packing stability. Short Cl⋯Cl contacts of 3.41 (2) Å are observed.

## Related literature

For applications of organoselenium compounds, see: Dinesh *et al.* (2007 ▶). For related structures, see: Fabiano *et al.* (2005 ▶); Fuller *et al.* (2010 ▶).
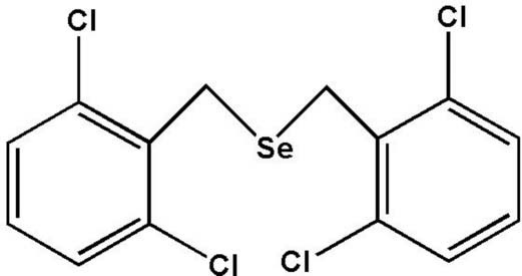


## Experimental

### 

#### Crystal data

C_14_H_10_Cl_4_Se
*M* *_r_* = 398.98
Monoclinic, 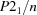

*a* = 8.1144 (5) Å
*b* = 12.2250 (5) Å
*c* = 15.3505 (9) Å
β = 102.479 (6)°
*V* = 1486.78 (14) Å^3^
*Z* = 4
Mo *K*α radiation
μ = 3.23 mm^−1^
*T* = 293 K
0.1 × 0.1 × 0.04 mm

#### Data collection

Agilent Xcalibur Sapphire3 Gemini ultra diffractometer
Absorption correction: multi-scan (*CrysAlis PRO*; Agilent, 2010 ▶) *T*_min_ = 0.659, *T*_max_ = 1.000
5405 measured reflections
2628 independent reflections
1902 reflections with *I* > 2σ(*I*)
*R*_int_ = 0.031

#### Refinement

*R*[*F* ^2^ > 2σ(*F* ^2^)] = 0.046
*wR*(*F* ^2^) = 0.127
*S* = 1.04
2628 reflections
172 parameters
H-atom parameters constrained
Δρ_max_ = 1.19 e Å^−3^
Δρ_min_ = −0.50 e Å^−3^

### 

Data collection: *CrysAlis PRO* (Agilent, 2010 ▶); cell refinement: *CrysAlis PRO*; data reduction: *CrysAlis PRO*; program(s) used to solve structure: *SHELXTL* (Sheldrick, 2008 ▶); program(s) used to refine structure: *SHELXTL*; molecular graphics: *SHELXTL*; software used to prepare material for publication: *publCIF* (Westrip, 2010 ▶).

## Supplementary Material

Crystal structure: contains datablock(s) global, I. DOI: 10.1107/S1600536812008318/nk2146sup1.cif

Structure factors: contains datablock(s) I. DOI: 10.1107/S1600536812008318/nk2146Isup2.hkl

Supplementary material file. DOI: 10.1107/S1600536812008318/nk2146Isup3.cml

Additional supplementary materials:  crystallographic information; 3D view; checkCIF report

## Figures and Tables

**Fig. 1.**
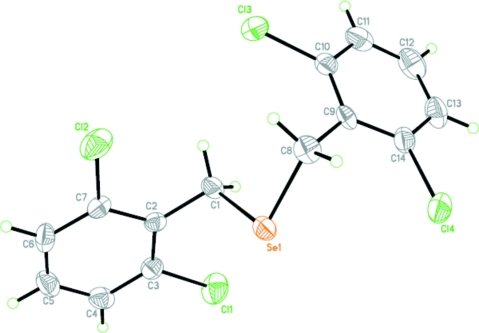
The molecular structure of the title compound. Displacement ellipsoids are drawn at the 30% probability.

## supplementary crystallographic information

### Comment

The interest in the chemistry of organoselenium compounds has increased remarkably in the last few decades due to their synthetic applications and biological activities(Dinesh *et al.*,2007). The title molecule, features a selenide bridge between two Dichlorobenzyl units. The dihedral angle between the two benzene rings is 107.9 (16)°. In the crystal, weak π–π intermolecular face-to-face aromatic interactions are observed [centroid-centroid distance between 6-Membered ring (C_2_, C_3_, C_4_, C_5_, C_6_, C_7_) and 6-Membered ring (C_9_, C_10_, C_11_, C_12_, C_13_, C_14_) = 3.885 (5) Å], providing some packing stability. Short Cl···Cl contacts of 3.41 (2) Å between Cl2 and Cl3 of adjacent molecules are also observed.

### Experimental

A solid mixture of sodium borohydride (0.38 g, 10 mmol) and elemental selenium (0.40 g, 5 mmol) is stirred in a two naked flask under argon and maintained at 20 °C using a water bath. Dropwise addition of anhydrous EtOH (1.40 g, 30 mmol) to this mixture favours the rapid evolvement of hydrogenand produces a white-grey solid. Addition of anhydrous DMF (10 mL) produces a red-brown solution, which slowly leads to a colourless one. 2,6-Dichlorobenzylchloride (10 mmol) is added dropwise to the solution of solution reported above. The resulting milky medium was stirred before hydrolysis and extraction with Et_2_O. The obtained organic layer was dried over MgSO_4_ overnight. The organic residue was further purified by silica gel column using dichloromethane as eluent, The solvent was evaporated and the solid residue was recrystallized from CH_3_Cl to give the product as yellow crystals (yield: 1.62 g, 80.5%).

### Refinement

Carbon-bound H atoms were positioned geometrically and treated as riding on their C atoms, with C—H distances of 0.93 Å(aromatic) and 0.97 Å(CH_2_) and were refined with *U*_iso_(H)=1.2Ueq(C). The height of the largest residual peak is 1.19, and the distance to the nearest non-H atom (se) is 1.07.

### Crystal data

**Table:**

C_14_H_10_Cl_4_Se	*F*(000) = 784
*M**_r_* = 398.98	*D*_x_ = 1.782 Mg m^−^^3^
Monoclinic, *P*2_1_/*n*	Mo *K*α radiation, λ = 0.7107 Å
*a* = 8.1144 (5) Å	Cell parameters from 1490 reflections
*b* = 12.2250 (5) Å	θ = 3.1–29.2°
*c* = 15.3505 (9) Å	µ = 3.23 mm^−^^1^
β = 102.479 (6)°	*T* = 293 K
*V* = 1486.78 (14) Å^3^	Plate, metallic pale yellow
*Z* = 4	0.1 × 0.1 × 0.04 mm

### Data collection

**Table:**

Agilent Xcalibur Sapphire3 Gemini ultra diffractometer	2628 independent reflections
Radiation source: Enhance (Mo) X-ray Source	1902 reflections with *I* > 2σ(*I*)
Graphite monochromator	*R*_int_ = 0.031
Detector resolution: 16.0288 pixels mm^-1^	θ_max_ = 25.0°, θ_min_ = 3.1°
ω scans	*h* = −9→9
Absorption correction: multi-scan (*CrysAlis PRO*; Agilent, 2010)	*k* = −11→14
*T*_min_ = 0.659, *T*_max_ = 1.000	*l* = −10→18
5405 measured reflections	

### Refinement

**Table:**

Refinement on *F*^2^	Primary atom site location: structure-invariant direct methods
Least-squares matrix: full	Secondary atom site location: difference Fourier map
*R*[*F*^2^ > 2σ(*F*^2^)] = 0.046	Hydrogen site location: inferred from neighbouring sites
*wR*(*F*^2^) = 0.127	H-atom parameters constrained
*S* = 1.04	*w* = 1/[σ^2^(*F*_o_^2^) + (0.060*P*)^2^] where *P* = (*F*_o_^2^ + 2*F*_c_^2^)/3
2628 reflections	(Δ/σ)_max_ < 0.001
172 parameters	Δρ_max_ = 1.19 e Å^−^^3^
0 restraints	Δρ_min_ = −0.50 e Å^−^^3^

### Special details

**Table:**

Geometry. All e.s.d.'s (except the e.s.d. in the dihedral angle between two l.s. planes) are estimated using the full covariance matrix. The cell e.s.d.'s are taken into account individually in the estimation of e.s.d.'s in distances, angles and torsion angles; correlations between e.s.d.'s in cell parameters are only used when they are defined by crystal symmetry. An approximate (isotropic) treatment of cell e.s.d.'s is used for estimating e.s.d.'s involving l.s. planes.
Refinement. Refinement of *F*^2^ against ALL reflections. The weighted *R*-factor *wR* and goodness of fit *S* are based on *F*^2^, conventional *R*-factors *R* are based on *F*, with *F* set to zero for negative *F*^2^. The threshold expression of *F*^2^ > σ(*F*^2^) is used only for calculating *R*-factors(gt) *etc*. and is not relevant to the choice of reflections for refinement. *R*-factors based on *F*^2^ are statistically about twice as large as those based on *F*, and *R*- factors based on ALL data will be even larger.

### Fractional atomic coordinates and isotropic or equivalent isotropic displacement parameters (Å^2^)

**Table:**

	*x*	*y*	*z*	*U*_iso_*/*U*_eq_	
Se1	0.89325 (7)	0.75838 (4)	0.18239 (3)	0.0556 (2)	
Cl3	0.52156 (18)	0.76390 (11)	−0.06014 (11)	0.0679 (4)	
Cl4	0.8752 (2)	1.05598 (11)	0.17334 (10)	0.0772 (5)	
Cl2	0.66481 (17)	0.48664 (13)	0.09733 (12)	0.0758 (5)	
Cl1	1.29563 (18)	0.64440 (12)	0.12621 (12)	0.0768 (5)	
C9	0.7067 (6)	0.9115 (3)	0.0528 (3)	0.0394 (11)	
C3	1.1568 (6)	0.5365 (4)	0.1322 (3)	0.0440 (11)	
C2	0.9848 (6)	0.5574 (3)	0.1111 (3)	0.0379 (10)	
C1	0.9110 (6)	0.6662 (3)	0.0799 (3)	0.0445 (12)	
H1A	0.7999	0.6561	0.0419	0.053*	
H1B	0.9818	0.7021	0.0452	0.053*	
C14	0.7921 (7)	1.0097 (4)	0.0657 (3)	0.0487 (12)	
C8	0.6873 (6)	0.8404 (4)	0.1287 (3)	0.0494 (12)	
H8A	0.5964	0.7888	0.1079	0.059*	
H8B	0.6552	0.8855	0.1743	0.059*	
C7	0.8824 (6)	0.4681 (4)	0.1202 (3)	0.0433 (11)	
C6	0.9468 (7)	0.3671 (4)	0.1470 (3)	0.0539 (14)	
H6	0.8745	0.3094	0.1519	0.065*	
C10	0.6395 (6)	0.8837 (4)	−0.0359 (3)	0.0432 (11)	
C5	1.1186 (8)	0.3511 (4)	0.1669 (4)	0.0593 (15)	
H5	1.1627	0.2824	0.1846	0.071*	
C11	0.6591 (7)	0.9485 (5)	−0.1069 (4)	0.0605 (14)	
H11	0.6120	0.9276	−0.1652	0.073*	
C13	0.8124 (8)	1.0760 (4)	−0.0042 (4)	0.0653 (16)	
H13	0.8693	1.1422	0.0071	0.078*	
C12	0.7480 (8)	1.0431 (5)	−0.0901 (4)	0.0700 (17)	
H12	0.7654	1.0860	−0.1373	0.084*	
C4	1.2247 (7)	0.4366 (4)	0.1604 (3)	0.0543 (14)	
H4	1.3411	0.4269	0.1750	0.065*	

### Atomic displacement parameters (Å^2^)

**Table:**

	*U*^11^	*U*^22^	*U*^33^	*U*^12^	*U*^13^	*U*^23^
Se1	0.0778 (5)	0.0396 (3)	0.0418 (3)	0.0097 (2)	−0.0041 (3)	0.0019 (2)
Cl3	0.0577 (9)	0.0589 (8)	0.0758 (10)	−0.0002 (6)	−0.0104 (7)	−0.0111 (7)
Cl4	0.1139 (14)	0.0446 (8)	0.0628 (10)	0.0014 (8)	−0.0035 (9)	−0.0108 (7)
Cl2	0.0427 (8)	0.0789 (10)	0.1042 (13)	−0.0092 (7)	0.0122 (8)	−0.0189 (9)
Cl1	0.0533 (9)	0.0711 (10)	0.1094 (13)	−0.0159 (7)	0.0250 (9)	0.0003 (9)
C9	0.044 (3)	0.031 (2)	0.044 (3)	0.015 (2)	0.014 (2)	0.006 (2)
C3	0.045 (3)	0.046 (3)	0.042 (3)	−0.003 (2)	0.011 (2)	−0.004 (2)
C2	0.039 (3)	0.042 (2)	0.030 (2)	0.000 (2)	0.004 (2)	−0.004 (2)
C1	0.049 (3)	0.043 (3)	0.039 (3)	0.002 (2)	0.006 (2)	0.010 (2)
C14	0.062 (3)	0.035 (3)	0.048 (3)	0.006 (2)	0.011 (3)	−0.001 (2)
C8	0.056 (3)	0.054 (3)	0.042 (3)	0.002 (2)	0.020 (2)	0.004 (2)
C7	0.038 (3)	0.046 (3)	0.045 (3)	−0.004 (2)	0.006 (2)	−0.010 (2)
C6	0.077 (4)	0.032 (3)	0.056 (3)	−0.008 (3)	0.021 (3)	−0.005 (2)
C10	0.041 (3)	0.042 (3)	0.045 (3)	0.007 (2)	0.005 (2)	0.000 (2)
C5	0.081 (4)	0.041 (3)	0.054 (3)	0.018 (3)	0.010 (3)	0.004 (2)
C11	0.067 (4)	0.068 (4)	0.044 (3)	0.023 (3)	0.007 (3)	0.003 (3)
C13	0.089 (5)	0.039 (3)	0.071 (4)	0.003 (3)	0.023 (3)	0.015 (3)
C12	0.090 (5)	0.065 (4)	0.062 (4)	0.013 (3)	0.032 (3)	0.027 (3)
C4	0.053 (3)	0.055 (3)	0.051 (3)	0.018 (3)	0.003 (3)	−0.006 (3)

### Geometric parameters (Å, º)

**Table:**

Se1—C1	1.965 (4)	C14—C13	1.382 (7)
Se1—C8	1.970 (5)	C8—H8A	0.9700
Cl3—C10	1.745 (5)	C8—H8B	0.9700
Cl4—C14	1.738 (5)	C7—C6	1.369 (7)
Cl2—C7	1.739 (5)	C6—H6	0.9300
Cl1—C3	1.749 (5)	C6—C5	1.375 (7)
C9—C14	1.379 (6)	C10—C11	1.385 (7)
C9—C8	1.490 (6)	C5—H5	0.9300
C9—C10	1.395 (6)	C5—C4	1.372 (7)
C3—C2	1.387 (6)	C11—H11	0.9300
C3—C4	1.371 (6)	C11—C12	1.359 (8)
C2—C1	1.494 (6)	C13—H13	0.9300
C2—C7	1.397 (6)	C13—C12	1.370 (8)
C1—H1A	0.9700	C12—H12	0.9300
C1—H1B	0.9700	C4—H4	0.9300
			
C1—Se1—C8	99.2 (2)	C2—C7—Cl2	118.5 (4)
C14—C9—C8	122.0 (4)	C6—C7—Cl2	118.9 (4)
C14—C9—C10	115.5 (4)	C6—C7—C2	122.6 (5)
C10—C9—C8	122.4 (4)	C7—C6—H6	120.1
C2—C3—Cl1	118.4 (4)	C7—C6—C5	119.8 (5)
C4—C3—Cl1	118.0 (4)	C5—C6—H6	120.1
C4—C3—C2	123.6 (5)	C9—C10—Cl3	119.6 (4)
C3—C2—C1	123.5 (4)	C11—C10—Cl3	117.6 (4)
C3—C2—C7	115.0 (4)	C11—C10—C9	122.8 (5)
C7—C2—C1	121.5 (4)	C6—C5—H5	120.1
Se1—C1—H1A	109.6	C4—C5—C6	119.9 (5)
Se1—C1—H1B	109.6	C4—C5—H5	120.1
C2—C1—Se1	110.3 (3)	C10—C11—H11	120.5
C2—C1—H1A	109.6	C12—C11—C10	119.0 (5)
C2—C1—H1B	109.6	C12—C11—H11	120.5
H1A—C1—H1B	108.1	C14—C13—H13	120.3
C9—C14—Cl4	120.0 (4)	C12—C13—C14	119.4 (5)
C9—C14—C13	122.6 (5)	C12—C13—H13	120.3
C13—C14—Cl4	117.4 (4)	C11—C12—C13	120.6 (5)
Se1—C8—H8A	108.8	C11—C12—H12	119.7
Se1—C8—H8B	108.8	C13—C12—H12	119.7
C9—C8—Se1	113.6 (3)	C3—C4—C5	119.1 (5)
C9—C8—H8A	108.8	C3—C4—H4	120.5
C9—C8—H8B	108.8	C5—C4—H4	120.5
H8A—C8—H8B	107.7		
